# The impact of tomato pomace silage on feed utilization, health status, milk yield, and composition in dairy cows

**DOI:** 10.5713/ab.250925

**Published:** 2026-06-01

**Authors:** Nirawan Gunun, Chatchai Kaewpila, Piyawit Kesorn, Thachawech Kimprasit, Metha Wanapat, Anusorn Cherdthong, Chanon Suntara, Waroon Khota, Pongsatorn Gunun

**Affiliations:** 1 Department of Animal Science, Faculty of Technology and Engineering, Udon Thani Rajabhat University, Udon Thani, Thailand; 2 Department of Animal Science, Faculty of Natural Resources, Rajamangala University of Technology Isan, Sakon Nakhon, Thailand; 3 Chakri Naruebodindra Medical Institute, Faculty of Medicine Ramathibodi Hospital, Mahidol University, Samut Prakan, Thailand; 4 Tropical Feed Resources Research and Development Center (TROFREC), Department of Animal Science, Faculty of Agriculture, Khon Kaen University, Khon Kaen, Thailand

**Keywords:** Functional Dairy Foods, Micronutrients, Thai Holstein, Tomato Waste

## Abstract

**Objective:**

The aim of this study was to evaluate the effect of the inclusion of tomato pomace silage (TPS) on feed intake, nutrient digestibility, blood parameters, immune response, milk yield, and composition in dairy cows.

**Methods:**

Sixteen Thai Holstein dairy cows with a mean body weight of 621±20 kg and 240±15 d in milk were randomly assigned to a completely randomized design. The TPS was added at 0%, 10%, 20%, and 30% dry matter (DM) in a fermented total mixed ration. Each cow was given the diet *ad libitum*.

**Results:**

The nutrient intake was similar among treatments except for ether extract intake, which increased linearly (p<0.01) with the addition of TPS. The inclusion of TPS at 20% and 30% linearly increased the digestibility of organic matter, crude protein, and ether extract (p≤0.01). The addition of TPS did not affect the blood chemicals or hematology except for monocytes, which increased cubically (p<0.05) when TPS were added. The immune function (IgA, IgM, and IgG) did not change with added TPS. There was no difference in milk yield and milk composition among treatments. Vitamin A and vitamin E in milk marginally elevated (p<0.01) by increasing levels of TPS. The inclusion of TPS increased the oleic acid (C18:1 cis-9), linoleic acid (C18:2 cis-9,12+trans-9,12), and α-linolenic acid (C18:3 cis-9,12,15) in milk (p<0.05). The unsaturated fatty acids and monounsaturated fatty acids in milk increased linearly (p<0.05) in cows fed TPS, but TPS did not impact polyunsaturated fatty acids.

**Conclusion:**

The inclusion of 30% (on a DM basis) of TPS in the diets enhanced the digestibility of nutrients, milk vitamins, and milk fat (unsaturated fatty acids) without affecting the feed intake, blood parameters, or milk yield in late-lactating dairy cows.

## INTRODUCTION

Dairy and milk products are often integral to nutritious and balanced human diets [1], thus serving as essential supplies of essential nutrients including protein, unsaturated fatty acids (UFAs), vitamins, and antioxidants. Consumers’ interest in healthy food and the demand for nutritious products have risen markedly in recent years. Consequently, it is essential to create innovative functional foods, especially milk and dairy foods, to satisfy these requirements. To enhance the profitability of dairy farms, it is essential to identify the most effective methods of converting feeds into nutritional milk for human consumption. Soybean meal (SBM) costs have recently risen but it is a principal protein source in diets due to its high protein content and excellent amino acid profile [2]. Research on alternative feeds is crucial to offer nutritionists and dairy farmers choices for formulating diets that sustain or enhance milk yield, milk composition, nutrient utilization, and cost-effectiveness via agro-industrial by-products [3].

Tomato pomace consists of the peel, seeds, and pulp that remain from the processing of juice, paste, and sauce. Tomato pomace thus wastes resources and can cause environmental pollution. Tomato pomace has a dry matter (DM) of 18.2%–26.0%, crude protein (CP) of 16.5%–21.7% DM, ether extract (EE) of 10.5%–13.4% DM, and neutral detergent fiber (NDF) of 52.2%–62.0% DM [4–6]. The tomato pomace primarily contains UFAs including 39.8%–48.2% linoleic acid (LA; C18:2 cis-9,12+trans-9,12), 10.1%–15.5% α-linolenic acid (ALA; C18:3 cis-9,12,15), and 10.6%–13.0% oleic acid (OA; C18:1 cis-9) [7]. Tomato pomace is an appropriate source of vitamin A, vitamin E, vitamin C, carotene, and lycopene. It contributes to the potential for antioxidants [4]. Tomato pomace can thus be a source of protein, fiber, and bioactive compounds in animal feeds. However, the higher moisture content of fresh tomato pomace by-products limits their utilization due to rapid spoilage [8].

Ensiling is a widely used procedure for preserving the nutritional value of high-moisture plant by-products [9]. It provides year-round ruminant feed [10,11]. Previous reports state that ensiling tomato pomace with whole plant corn or apple pomace enhances the quality of silage and feed utilization while maintaining milk yield and composition in dairy cows [5,12]. Tuoxunjiang et al. [4] reported that the inclusion of tomato pomace silage (TPS) at 10% as a partial replacement of whole plant corn silage resulted in improved feed intake; vitamin A, E, and C levels in milk; antioxidant capacity; and immune status without affecting milk production and composition in early-lactating dairy cows. Hence, TPS has potential value in ruminant diets.

The effects of the inclusion of TPS in dairy cows’ feed intake, digestibility, health status, milk production, milk vitamins, and fatty acid profiles are limited. No current research has been conducted at practical levels (10% to 30% in fermented total mixed ration, FTMR). We hypothesize that the inclusion of TPS at suitable levels for ruminants could improve their health indicators, milk vitamins, and milk UFA without having a negative effect on feed intake, nutrient digestibility, or milk production. Therefore, the objective of this study is to examine the impact of TPS on the feed utilization, blood parameters, immune response, milk yield, milk vitamins, and fatty acid profiles in dairy cows.

## MATERIALS AND METHODS

### Preparation of tomato pomace silage

TP was obtained from a local paste factory in Nong Khai, Thailand. Fresh tomato pomace (without any additive) was then stored in bunker silos (length×width×height 25×5×3 m) made with concrete walls and floors and covered with a black polyethylene sheet and tires with air temperature of 25^o^C–36^o^C. After 30 days of ensiling, the silos were opened to measure the pH of the silage using a portable pH meter (FiveGo, Mettler-Toledo GmbH, Greifensee). The organic acids were evaluated using gas chromatography (Nexis GC-2030, Shizuku Co.). The samples were analyzed for DM (method 930.15), EE (method 920.39) (ST 243 Soxtec; FOSS), and ash (method 942.05) (Vulcan 3-550; DENTSPLY International) [13]. The CP content was determined via a N analyzer (828 Series; LECO). The amount of NDF (method 2002.04) and acid detergent fiber (ADF) (method 973.18) was evaluated via a fiber analyzer (ANKOM 200; ANKOM Technology) [14]. The fatty acid profiles were evaluated using gas chromatography (GC 8890; Agilent Technologies) with the Cristie [15] method. The diets were formulated to be isonitrogenous containing 14.0%–14.5% CP on a DM basis as well as isocaloric with total digestible nutrients (TDN) levels ranging from 71.2% to 72.8% in accordance with recommended nutrient requirements [16].

### Animals, treatments, and experimental design

This study was conducted at Racha Dairy Farm under the administration and supervision of the Dairy Farming Promotion Organization (DPO) in the northeastern region of Thailand. Sixteen late-lactating healthy Thai Holstein dairy cows were arranged in a completely randomized design pattern to receive four treatments. The cows had a mean body weight (BW) of 621±20 kg, 240±15 d in milk, and produced 10.8±1.0 kg of milk each day. Dietary treatments were based on TPS levels of 0%, 10%, and 20% in FTMR diets (Table 1).

The feed ingredients were mixed thoroughly and then packed using polyethylene stretch film using a silage baler machine (Taizy Agro-Machinery) at approximately 50 kg/baler. The silages were stored at room temperature and were then opened after a fermentation period of 30 d before feeding to animals. The cows were fed the diets *ad libitum* at 08:00 h, 12:00 h, and 17:00 h every day. The experiment was conducted over a 60-d period with the initial 14 d serving as an adaptation period. The cows were kept in separate enclosures with access to clean drinking water.

### Data collection and sampling procedures

Feed consumption was measured every day by weighing the feed provided and the remainder that was not eaten. Feces from each animal was collected for evaluation of digestibility over the last five days of the trial (56 d to 60 d). Rectal sampling was used to obtain approximately 500 g of fresh fecal samples in the morning (07:30 h) and afternoon (16:30 h). Two consecutive samples were mixed, and the composite was thereafter stored in the refrigerator at 4°C. The samples, comprising feed, refusals, and feces, were dried at 60°C in a hot air oven and ground using a 1-millimeter screen with a Cyclotech Mill (Tecator). The samples were previously analyzed for DM, EE (ST 243 Soxtec; FOSS), and ash (Vulcan 3-550; DENTSPLY International) [13]. The CP content was measured via a N analyzer (828 Series; LECO). The concentrations of NDF and ADF were evaluated using the fiber analyzer (ANKOM 200; ANKOM Technology) [14]. To examine fatty acid profiles in feed utilizing gas chromatography (GC 8890; Agilent Technologies) [15]. Nutrient digestibility was assessed utilizing acid-insoluble ash (AIA) as an internal marker [17].

Cows were milked twice daily at 07:00 h and 16:00 h, and the daily milk yield of each animal was recorded. On the final five days of the experiment (56 to 60 d), they collected a daily milk sample totaling 100 mL: 60 mL from the morning and 40 mL from the afternoon. Samples were refrigerated at 4°C. The milk samples were analyzed for fat, protein, lactose, total solids, and solids other than fat via infrared techniques with the Milko-Scan 33 (Foss Electric). The somatic cell count (SCC) used a Fossomatic 5000 (Foss Electric). The milk fatty acid profiles were analyzed using gas chromatography (GC 8890; Agilent Technologies) via the Cristie [15] method. The measure of vitamins in milk includes vitamin A (retinol) [18], vitamin C (ascorbic acid) [19], and vitamin E (α-tocopherol) [20] using high-performance liquid chromatography (HPLC; Agilent Technologies).

At 4 h post-feeding on the final day of the experiment, freshly drawn blood samples (approximately 10 mL) were obtained from the jugular vein of each cow. A chemical analyzer (Mindray BS-600; Mindray) was used to evaluate blood urea nitrogen (BUN), glucose, total protein, cholesterol, and triglycerides. The hemoglobin, hematocrit, red blood cell (RBC), neutrophils, lymphocytes, monocytes, and eosinophils of the blood were measured using a hematology analyzer (Mindray BC-3000 Plus; Mindray). In addition, the blood sample was assessed for immunoglobulins (IgA, IgG, and IgM) utilizing the nephelometric method (Mispa-i3; Agappe Diagnostics).

### Statistical analysis

Data were analyzed according to the MIXED procedure of SAS [21]. The following model was used to analyze milk yield through repeated measurements:


(1) $$Y_{ijk}=\mu +\alpha_{i}+\beta_{j}+{\left(\alpha \beta \right)}_{ij}+\delta_{ik}+{ɛ}_{ijk},$$

Here, *Yijk* represents the dependent variable, *μ* is the overall mean, *α_i_* is the fixed effect of treatment (where *i* = 1 to 4), *β_j_* indicates the fixed effect of milk collection time (with *j* = 1 to 4, corresponding to the time of 0–15 d, 16–30 d, 31–45 d, and 46–60 d), (*αβ*)*_ij_* is the fixed interaction effect between *α_i_* and *β_j_*, *δ_ik_* is the random effect associated with the individual cow within *α_i_*, and *ɛ_ijk_* is the residual error. Time is defined as a repeating measurement. The covariance structure that was most suitable for repeated measures was determined via the least Bayesian information criterion (BIC) value. The analysis in this model initially included lactation, day in milk, and initial BW of the dairy cows as covariates. However, they were removed from the final analysis because they were not statistically significant (p>0.05). The LS-means were separated with the PDIFF tool. In addition, treatment means for increasing TPS levels were subjected to orthogonal polynomial analysis to test for linear, quadratic, and cubic effects. Statistically significant variations were accepted at p<0.05.

## RESULTS

### Chemical composition of diets

The FTMR diets had a CP of 14.0%–14.5% (Table 1). The inclusion of TPS in FTMR slightly decreased DM and NDF content. However, the increasing levels of TPS in FTMR led to an increase in the EE content. The feed costs of FTMR with TPS at 0%, 10%, 20%, and 30% are 16.4, 13.4, 10.8, and 8.8 USD/100 kg DM, respectively. The TPS contains 16.5% DM, 18.1% CP, 10.6% EE, and 65.9% NDF (Table 2). TPS had high UFA content including OA (20.02%), LA (55.63%), and ALA (2.34%).

### Feed intake and nutrient digestibility

The inclusion of TPS did not affect the intake of DM, organic matter (OM), CP, NDF, or ADF, while EE intake was increased linearly (p<0.01) with increasing levels of TPS (Table 3). The digestibility of DM, NDF, and ADF was similar among treatments. However, the digestibility of OM, CP, and EE increased linearly (p≤0.01) with the addition of TPS at 20% and 30%.

### Blood chemicals and hematological parameters

The concentrations of glucose, BUN, total protein, cholesterol, and triglyceride were similar among treatments (Table 4). The inclusion of TPS did not affect hemoglobin, hematocrit, RBC, neutrophils, lymphocytes, or eosinophils. However, the inclusion of TPS at 20% and 30% resulted in a cubic increase in monocyte levels (p<0.01).

### Immune response

The addition of varying amounts of TPS to dairy cows did not affect concentrations of IgA, IgM, and IgG (Table 5).

### Milk production and composition

The inclusion of TPS did not impact milk yield (Table 6). Milk fat, protein, lactose, solids-not-fat, total solids, and SCC did not change in cows fed TPS. Vitamin C (ascorbic acid) levels were not detectable in milk from cows fed TPS. The inclusion of TPS resulted in a linear increase (p<0.01) in both vitamin A (retinol) and vitamin E (a-tocopherol) concentrations in milk. The income over feed cost (IOFC) was similar among treatments.

### Fatty acid profile in milk

The inclusion of TPS resulted in a significant increase in lauric acid (C12:0), pentadecanoic acid (C15:0), and cis-10-pentadecenoic acid methyl ester (C15:1, cis-10), while the levels of lignoceric acid (C24:0) decreased (p<0.05) with the addition of TPS at 10% and 20% (Table 7). Adding TPS increased the concentrations of OA, LA, and ALA. The inclusion of TPS at 30% had a higher concentration of UFA and monounsaturated fatty acid (MUFA) in milk (p<0.05), but the saturated fatty acid (SFA) and polyunsaturated fatty acid (PUFA) in milk were similar among treatments.

## DISCUSSION

The levels of CP and EE in TPS were 18.1% and 10.6%, respectively, which are similar to previous studies [22]. The NDF content (65.9%) of TPS was similar to the reports of Wu et al. [23] (NDF 69.4%), but higher than Abdollahzadeh et al. [5], which found that the NDF content was at 57.4%. This outcome may have been caused by the industry’s processing of tomatoes; the ratios of peel, seed, and pulp; and ensiling time—these variables can impact NDF levels in the TPS. The primary fatty acids found in tomato pomace are LA, OA, palmitic acid (C16:0), and ALA. The study’s findings are consistent with earlier research on fresh tomato pomace [24]. TPS’s chemical composition in this study indicates that cows can use it as a source of roughage, protein, and lipids—especially UFA. We found that TPS has a pH of 4.46, which is similar to the results of previous studies that reported a pH of 4.60 for TPS after 30 days of ensiling [23]. Lactic acid facilitates prolonged preservation of silage by establishing a low pH environment that inhibits the proliferation of undesirable microbes [25,26].

The added TPS (10% to 30%) in FTMR had no effect on the DM intake in late-lactating dairy cows. However, Tuoxunjiang et al. [4] reported that the inclusion of TPS at 10% in TMR improved DM intake in early-lactating cows. These results may be attributed to variations in lactation stages, the ingredients used in the diets, or the nutritional requirements of TMR—these variables could all impact feed intake in dairy cows. Adding TPS into the diet can improve the intake and digestion of EE. The results may be attributed to increasing levels of TPS in the diets, which have been shown to slightly increase the EE content, thus improving EE intake and digestibility in cows. In addition, increasing levels of TPS increased the CP’s digestibility. In contrast to previous studies, we found that corn ensiled with tomato pomace at a ratio of 88:12 had no effect on the digestibility of CP in dairy cows [12]. These results may be attributed to two possible explanations: First, there is reduced CP digestibility of diets formulated with corn silage versus those with a mixture of CS and other silage. The nitrogen in the maize grain from the silage passes through the rumen and enters the lower tract undigested leading to increasing fecal N secretion thus lowering CP digestibility [27]. Second, during the ensiling, the microbial-induced degradation of protein in tomato pomace to peptide or amino acid also leads to increased digestibility in the rumen and small intestine of animals. Moreover, the increased digestibility of CP and EE results in higher OM digestibility when TPS are included at 20% and 30% in the diets.

Chemical blood parameters play a crucial role in assessing animal metabolic status, health, and dairy product quality. Cows fed TPS had no effect on the blood chemistry including glucose, BUN, and total protein. Similarly, Tuoxunjiang et al. [4] reported that the inclusion of TPS in the cows’ diet did not significantly affect the blood chemistry. Moreover, tomato exhibited hypocholesterolemic effects by inhibiting 3-hydroxy-3-methylglutaryl coenzyme. The activity of reductase (HMGCR) functions as the rate-limiting enzyme in cholesterol metabolism [28]. The addition of TP led to a reduction in blood cholesterol and triglyceride levels in chickens [29]. In contrast, we found that the cholesterol and triglycerides in blood did not change when cows received TPS. These findings agreed with prior investigations, which showed that the inclusion of TP had no effect on the cholesterol and triglyceride in dairy cattle [4,30]. The difference in digestive tract, metabolism, and nutrient use efficiency between non-ruminants and ruminants might impact the concentration of blood cholesterol and triglycerides.

Monocytes serve an important role in regulating the main components of the innate immune response [31]. β-carotene can enhance cell-mediated immune responses by increasing the cell surface expression of major histocompatibility complex (MHC) class II molecules in animals [32]. Here, we found that adding TPS increased the concentration of monocytes. This result may be due to the inclusion of TPS containing β-carotene, which can enhance cell-mediated immune responses by increasing the number of blood monocytes.

The immune system and other cells in animals depend on their dietary state and nutrient metabolism [33]. Vitamins A, C, and E substantially impact the immune system function through multiple mechanisms [34]. The TP is a natural source of vitamins C, E, and A (retinol and β-carotene). Prior studies have indicated that incorporating 10% TPS into diets increased the levels of immunoglobulins (IgA, IgM, and IgG) before and after 14 d of calving in dairy cows [4]. In contrast with our study, the inclusion of TPS at 10% to 30% did not alter IgA, IgM, or IgG concentrations in late-lactating dairy cows. Immune system functions are reduced during the weeks before and after calving, thus leading to a high incidence of infectious diseases in cows [35]. Early-lactating cows could have a more acute inflammatory response relative to mid- or late-lactating cows. Therefore, the inclusion of vitamins and antioxidants in TPS may have had a greater impact on the immune functions of early-lactating cows in previous studies versus the late-lactating cows in our study.

The inclusion of TPS did not affect milk yields when fed to cows. In agreement with our findings, adding TPS in the diets did not alter milk production in early-lactating dairy cows (milk yield 24 kg/hd/d) [4]. This study uses late-lactating cows with low milk yield (9.9–11.7 kg/hd/d) with a small sample size. Hence, further research is needed to investigate TPS on milk production and other parameters in high-producing dairy cows with a larger sample size. Milk fat constitutes the primary energy component of milk and plays an essential function in the effectiveness of dairy production. Our findings showed that the addition of TPS at 10% to 30% in FTMR had no effect on milk fat. This result agrees with previous studies, which found that the inclusion of corn plus TPS did not influence the milk fat in dairy cows [12]. This indicated that using TPS as a roughage source has the potential to be used in FTMR without affecting the milk fat synthesis in dairy cattle.

Milk and dairy products have been recognized as credible sources of vitamins. Enhancing the vitamin content in ruminant milk is likely attainable mostly through modifications in dietary composition [36]. Vitamin A refers to a class of fat-soluble compounds exhibiting retinol activity—primarily comprising retinol and retinyl esters, as well as provitamin A carotenoids, especially β-carotene, which serve as precursors to vitamin A and are predominantly sourced from plants [37]. Vitamin A enhances the defense against oxidative stress [38]. Feed ingredients in the diets appear to have a major influence on the amount of vitamin A in cow’s milk. The TPS has vitamin A (retinol) at 23,600 IU/kg and b-carotene at 90 mg/kg [4]. The addition of TPS thus increases the concentration of vitamin A in milk. These findings agree with previous investigations that have found that including TPS at 10% in the TMR of cows increases the vitamin A content of milk [4]. Vitamin E, also commonly referred to as tocopherol, is an effective antioxidant that protects cells against free oxidative damage and supports healthy immunity [39]. Tomato waste is a major source of vitamin E (α-tocopherol)—mainly in tomato seeds (0.56–32.7 mg/100 g) [22]. The concentrations of vitamin E in milk were increased with increasing levels of TPS. Similarly, Tuoxunjiang et al. [4] reported that the inclusion of TPS increased the concentration of milk vitamin E in cows. This data indicated that the milk in cows receiving TPS is a key functional food—particularly as a source of vitamins for humans.

Milk fatty acids are produced either by fatty acids absorbed from the bloodstream or through de novo synthesis in the mammary glands of animals [40]. The agro-industrial by-products from tomato processing contain higher levels of OA, LA, and ALA, thus providing suitable sources of UFA for ruminants. Rumen microorganisms can convert LA and ALA to stearic acid (C18:0) when ruminants ingest C18 UFA in their feed; this process is called biohydrogenation [33]. The milk fat of cows fed TPS had increased concentrations of OA, LA, and ALA. This results in the plausibility that the initial higher concentration of free UFAs, specifically C18:2 and C18:3, inhibits some steps in the biohydrogenation process or slows the rate of biohydrogenation within the rumen [41]. Hence, increasing the concentrations of biohydrogenation intermediates leads to an increase in C18 UFA in milk. These results agree with previous studies indicating that adding tomato pomace to the diet increased OA, LA, and ALA in the milk of dairy goats and sheep [42–44].

The goal of nutrient manipulation during milk fat synthesis is to enhance the concentration of PUFA. Previous studies indicated that the addition of TP increased the PUFA in the milk of goats and sheep [43,45]. The fatty acids in by-products that are accessible to biohydrogenating microbes increases biohydrogenation intermediates, whereas the other fatty acids may remain protected, thus increasing the availability of PUFA absorption in the small intestine [40]. Another reason is that the high levels of PUFA in the TP leads to a higher concentration of ALA when fed to animals, which is the main source of PUFA in milk fat [44]. In contrast to our study, we found that the inclusion of TPS did not affect the milk PUFA in dairy cows. Adding TPS at 10% to the diets increased the milk ALA to 0.011% of total fatty acids versus the control group, which had 0.09% of total fatty acids; however, this increase was minimal versus prior studies of goats and sheep [42,44,45], and it may not have altered the concentration of PUFA in the milk of cows in our study. Nevertheless, the UFA and MUFA were slightly increased at higher levels of TPS. This study suggests that the inclusion of TPS in FTMR feeds could be an option to improve the UFA of cows’ dairy products as functional foods for humans.

## CONCLUSION

In conclusion, 30% (on a DM basis) of TPS in the diets improved the nutrient digestibility, milk vitamins, and C18 UFA in milk fat—particularly LA and ALA. However, it has no effect on the feed intake, blood chemicals, hematological parameters, immune response, or milk yields in dairy cows. Therefore, TPS has the potential to be used in diets and reduce feed costs for late-lactating dairy cows. But further studies are required to evaluate the effect of the addition of TPS on digestibility, blood parameters, milk production, and milk nutritional composition during the early lactation of high-producing dairy cows.

## Figures and Tables

**Table 1:** Ingredients and chemical composition of fermented total mixed ration (FTMR) and tomato pomace silage (TPS)

Items	TPS (%DM)			

	0	10	20	30
Ingredient (kg DM)				
Corn silage	52.5	42.6	33.3	22.2
Tomato pomace silage	0.0	10.0	20.0	30.0
Cassava chip	17.4	18.5	19.5	21.5
Soybean meal	14.8	12.3	9.3	6.3
Rice bran	8.7	9.0	9.3	9.8
Rice straw	2.7	3.7	4.7	6.2
Minerals1)	2.3	2.3	2.3	2.3
Vitamins2)	0.9	0.9	0.9	0.9
Urea	0.6	0.6	0.6	0.6
Sulfur	0.1	0.1	0.1	0.1
Chemical composition				
DM (%)	42.6	41.2	35.4	34.9
Organic matter (%DM)	90.7	90.8	92.1	91.8
Crude protein (%DM)	14.0	14.5	14.3	14.1
Ether extract (%DM)	2.0	3.7	4.1	5.7
Neutral detergent fiber (%DM)	47.8	49.8	48.1	47.4
Acid detergent fiber (%DM)	19.8	21.7	22.3	24.1
Ash (%DM)	9.3	9.2	7.9	8.2
TDN3)	72.8	72.4	71.8	71.2
Feed cost (USD/100 kg)	16.4	13.4	10.8	8.8

1) Contains per kilogram mineral: 107.78 g Na; 93.72 g Ca; 46.86 g P; 18.55 g S; 8.24 g Mn; 7.49 g Zn; 3.37 g Mg; 1.17 g Cu; 0.15 g Co; 0.04 I; 0.02 Se; 0.01 g K.

2) Contains per kilogram vitamin: 5,000,000 IU vitamin A; 1,000,000 IU vitamin D; 11,000 IU vitamin E.

3) Calculate value.

DM, dry matter; TDN, total digestible nutrients.

**Table 2:** Chemical composition and fatty acid profile of tomato pomace silage (TPS)

Item	TPS
Chemical composition	
Dry matter (DM) (%)	16.5 (±0.26)
Organic matter (%DM)	94.5 (±0.25)
Crude protein (%DM)	18.1 (±0.13)
Ether extract (%DM)	10.6 (±0.12)
Neutral detergent fiber (%DM)	65.9 (±0.23)
Acid detergent fiber (%DM)	55.2 (±0.62)
Ash (%DM)	5.5 (±0.25)
Fatty acid (% of total fatty acid)	
C16:0	13.7 (±0.05)
C16:1	0.24 (±0.08)
C17:0	0.14 (±0.04)
C17:1	0.19 (±0.03)
C18:0	4.92 (±0.21)
C18:1 cis-9 (OA)	20.02 (±0.24)
C18:2 cis-9,12+trans-9,12 (LA)	55.63 (±0.17)
C18:3 cis-9,12,15 (ALA)	2.34 (±0.14)
C20:0	0.70 (±0.05)
C20:1	0.11 (±0.03)
C20:2	0.04 (±0.01)
C22:0	0.40 (±0.05)
C24:0	0.19 (±0.03)
Silage quality	
pH	4.46 (±0.01)
Lactic acid (g/kg DM)	2.43 (±0.13)
Acetic acid (g/kg DM)	99.89 (±0.35)
Propionic acid (g/kg DM)	55.84 (±0.24)
Butyric acid (g/kg DM)	8.74 (±0.11)

OA, oleic acid; LA, linoleic acid; ALA, a-linolenic acid.

**Table 3:** Effect of tomato pomace silage (TPS) on nutrient intake and digestibility in dairy cows

Item	TPS (%DM)				SEM	Trt	Contrast		
	
	0	10	20	30			L	Q	C
Nutrient intake (kg/d)									
DM	12.2	12.4	11.3	12.2	0.84	0.81	0.77	0.65	0.39
Organic matter	11.1	11.3	10.4	11.2	0.76	0.85	0.89	0.67	0.44
Crude protein	1.7	1.8	1.6	1.7	0.11	0.59	0.72	0.81	0.20
Ether extract	0.3^c^	0.5^b^	0.5^b^	0.7^a^	0.03	<0.01	<0.01	0.58	0.10
Neutral detergent fiber	5.8	6.2	5.3	5.1	0.37	0.24	0.10	0.51	0.27
Acid detergent fiber	2.4	2.7	2.5	2.9	0.16	0.15	0.08	0.55	0.13
Digestibility coefficients (%)									
DM	54.2	52.9	54.3	55.3	2.57	0.92	0.69	0.64	0.78
Organic matter	60.4^bc^	57.8^c^	66.9^ab^	69.0^a^	2.80	0.04	0.01	0.40	0.16
Crude protein	58.0^b^	58.3^b^	64.2^ab^	68.2^a^	2.90	0.03	0.01	0.52	0.58
Ether extract	60.6^b^	55.9^b^	73.4^a^	76.9^a^	3.76	<0.01	<0.01	0.30	0.06
Neutral detergent fiber	41.4	44.3	43.8	41.1	3.16	0.84	0.92	0.39	0.95
Acid detergent fiber	35.7	39.3	36.7	35.0	1.81	0.36	0.57	0.16	0.39

a–c Values in the same row with different superscripts differ according to p<0.05.

DM, dry matter; SEM, standard error of the mean; L, linear; Q, quadratic; C, cubic.

**Table 4:** Effect of tomato pomace silage (TPS) on blood chemistry and hematology in dairy cows

Item	TPS (%DM)				SEM	Trt	Contrast		
	
	0	10	20	30			L	Q	C
Glucose (mg%)	49.3	49.3	49.0	47.3	2.54	0.93	0.59	0.74	0.91
BUN (mg/dL)	15.5	14.3	10.5	14.5	1.47	0.14	0.32	0.10	0.14
Total protein (g/dL)	8.1	7.7	7.3	7.9	0.28	0.08	0.25	0.06	0.29
Cholesterol (g/dL)	127.5	120.5	130.0	127.8	15.34	0.97	0.88	0.88	0.69
Triglyceride (mg/dL)	11.5	10.5	11.5	12.0	1.96	0.95	0.78	0.70	0.76
Hemoglobin (g/L)	8.6	9.2	9.0	8.8	0.40	0.72	0.78	0.31	0.68
Hematocrit (%)	28.3	30.2	28.9	28.4	1.24	0.68	0.86	0.33	0.49
Red blood cell (10^12^/L)	6.4	6.7	6.6	6.7	0.31	0.88	0.63	0.66	0.67
Neutrophils (%)	24.0	31.8	26.3	33.5	3.21	0.15	0.11	0.92	0.09
Lymphocytes (%)	67.3	57.0	64.8	52.8	4.61	0.15	0.10	0.85	0.09
Monocytes (%)	6.3^b^	4.3^b^	9.5^a^	7.8^ab^	0.99	0.02	0.04	0.90	<0.01
Eosinophils (10^9^/L)	2.5	7.0	4.8	6.0	1.47	0.21	0.23	0.29	0.14

a,b Values in the same row with different superscripts differ according to p<0.05.

DM, dry matter; SEM, standard error of the mean; L, linear; Q, quadratic; C, cubic; BUN, blood urea nitrogen.

**Table 5:** Effect of tomato pomace silage (TPS) on immune response in dairy cows

Item	TPS (%DM)				SEM	Trt	Contrast		
	
	0	10	20	30			L	Q	C
IgA (mg/dL)	81.3	77.0	75.8	81.8	3.27	0.49	0.98	0.14	0.77
IgM (mg/dL)	43.3	41.3	44.3	48.5	2.85	0.37	0.17	0.29	0.77
IgG (mg/dL)	552.3	571.0	577.8	581.5	30.37	0.90	0.49	0.80	0.95

DM, dry matter; SEM, standard error of the mean; L, linear; Q, quadratic; C, cubic.

**Table 6:** Effect of tomato pomace silage (TPS) on milk yield and milk composition in dairy cows

Item	TPS (%DM)					p-value1)			Contrast		
			
	0	10	20	30	SEM	Trt	Time	Trt×Time	L	Q	C
Production (kg/d)											
Milk yield	11.7	10.7	11.0	9.9	1.26	0.79	<0.01	0.27	0.38	0.98	0.65
4% FCM	13.2	11.5	12.0	12.7	1.77	0.90	<0.01	0.44	0.91	0.52	0.79
Milk composition (%)											
Fat	4.7	4.5	4.6	5.3	0.33	0.43	-	-	0.26	0.19	0.87
Protein	3.3	3.6	3.2	3.4	0.28	0.67	-	-	0.79	0.87	0.25
Lactose	4.3	4.3	4.4	4.1	0.09	0.23	-	-	0.22	0.24	0.16
Solids-not-fat	8.9	9.0	8.8	8.8	0.26	0.87	-	-	0.68	0.81	0.52
Total solids	13.6	13.4	13.3	13.9	0.45	0.80	-	-	0.62	0.42	0.71
Vitamin A (retinol) (mg/100 mL)	11.4^b^	19.4^b^	20.0^b^	38.3^a^	3.19	<0.01	-	-	<0.01	0.14	0.11
Vitamin C (ascorbic acid) (mg/100 g)	ND	ND	ND	ND	-	-	-	-	-	-	-
Vitamin E (α-tocopherol) (mg/kg)	1.4^b^	2.1^ab^	2.2^a^	2.7^a^	0.21	0.01	-	-	<0.01	0.72	0.35
Somatic cell counts (10^5^ cell/mL)	2.1	3.3	4.4	2.3	0.46	0.46	-	-	0.75	0.21	0.58
Income over feed cost (USD/kg)	5.2	5.0	5.6	5.1	0.75	0.94	-	-	0.94	0.87	0.87

1) Trt = Treatment; Time = 0–15 d, 16–30 d, 31–45 d, and 46–60 d.

a,b Values in the same row with different superscripts differ according to p≤0.01.

DM, dry matter; SEM, standard error of the mean; L, linear; Q, quadratic; C, cubic; FCM, fat-collected milk; ND, not detected.

**Table 7:** Effect of tomato pomace silage (TPS) on fatty acid profiles in milk of dairy cows

Fatty acid (% of total fatty acid)	TPS (%DM)				SEM	Trt	Contrast		
	
	0	10	20	30			L	Q	C
C4:0	2.2	1.9	2.0	1.9	0.18	0.58	0.35	0.62	0.37
C6:0	1.7	1.6	1.6	1.4	0.13	0.30	0.11	0.69	0.35
C8:0	1.0	0.9	0.9	0.8	0.07	0.19	0.08	0.39	0.30
C10:0	2.0	1.9	1.9	1.6	0.17	0.34	0.14	0.48	0.41
C11:0	0.1	0.1	0.1	0.1	0.05	0.95	0.64	0.97	0.79
C12:0	0.8^b^	2.2^a^	2.2^a^	1.9^a^	0.32	0.03	0.04	0.03	0.50
C13:0	0.3	0.2	0.2	0.4	0.14	0.54	0.44	0.26	0.64
C14:0	10.2	10.6	10.3	10.1	0.60	0.91	0.82	0.60	0.69
C15:0	0.8^b^	1.0^a^	0.8^b^	0.9^ab^	0.04	0.03	0.45	0.06	0.02
C16:0	30.3	34.0	30.3	28.7	1.39	0.10	0.20	0.08	0.15
C17:0	1.1	1.2	0.7	1.7	0.41	0.42	0.52	0.31	0.24
C18:0	9.2	8.8	9.5	8.2	1.52	0.94	0.75	0.79	0.68
C14:1 cis-9	0.8	1.2	0.9	1.1	0.11	0.10	0.26	0.25	0.06
C15:1 cis-10	0.2^b^	0.4^a^	0.3^a^	0.3^a^	0.03	0.01	0.06	0.02	0.08
C16:1 cis-9	1.4	2.2	1.4	1.8	0.21	0.06	0.56	0.41	0.05
C17:1 cis-10	0.4	0.3	0.6	0.6	0.26	0.79	0.49	0.80	0.50
C18:1 cis-9 (OA)	20.2^b^	25.1^ab^	24.6^ab^	28.2^a^	2.41	0.04	0.03	0.79	0.38
C18:2 cis-9,12+trans-9,12 (LA)	1.1^b^	1.6^a^	1.4^b^	1.4^b^	0.10	0.03	0.33	0.02	0.12
C18:3 cis-9,12,15 (ALA)	0.09^b^	0.11^a^	0.10^b^	0.10^b^	0.01	0.04	0.84	0.03	0.18
C20:2	1.1	0.3	0.6	0.8	0.27	0.28	0.63	0.12	0.29
C20:3	0.09^b^	0.14^a^	0.15^a^	0.14^a^	0.01	0.03	0.01	0.08	0.80
C20:4	0.07	0.02	0.03	0.04	0.02	0.44	0.45	0.20	0.49
C20:5 (EPA)	0.12	0.08	0.10	0.11	0.05	0.93	0.99	0.58	0.73
C24:0	0.3^a^	0.1^b^	0.1^b^	0.2^a^	0.04	0.04	0.16	0.02	0.23
SFA	63.1	64.4	61.6	57.7	2.12	0.20	0.10	0.26	0.74
UFA	25.5^b^	31.4^ab^	32.1^ab^	34.5^a^	2.38	0.04	0.02	0.48	0.55
MUFA	22.9^b^	29.1^ab^	30.0^ab^	31.9^a^	2.55	0.03	0.02	0.43	0.59
PUFA	2.6	2.3	2.5	2.5	0.28	0.87	0.97	0.50	0.64

a,b Values in the same row with different superscripts differ according to p<0.05.

DM, dry matter; SEM, standard error of the mean; L, linear; Q, quadratic; C, cubic; OA, oleic acid; LA, linoleic acid; ALA, a-linolenic acid; EPA, eicosapentaenoic acid; SFA, saturated fatty acids; UFA, unsaturated fatty acids; MUFA, monounsaturated fatty acids; PUFA, polyunsaturated fatty acid.
